# A SERS Composite Hydrogel Device for Point-of-Care Analysis of Neurotransmitter in Whole Blood

**DOI:** 10.3390/bios13060611

**Published:** 2023-06-02

**Authors:** Lei Wu, Xuefeng Liu, Shenfei Zong, Zhuyuan Wang, Yiping Cui

**Affiliations:** Advanced Photonics Center, School of Electronic Science and Engineering, Southeast University, Nanjing 210096, China; lwu@seu.edu.cn (L.W.); 220201515@seu.edu.cn (X.L.); sfzong@seu.edu.cn (S.Z.)

**Keywords:** surface enhanced Raman spectroscopy (SERS), hydrogel, neurotransmitter, point-of-care test

## Abstract

Point-of-care analysis of neurotransmitters in body fluids plays a significant role in healthcare improvement. Conventional approaches are limited by time-consuming procedures and usually require laboratory instruments for sample preparation. Herein, we developed a surface enhanced Raman spectroscopy (SERS) composite hydrogel device for the rapid analysis of neurotransmitters in whole blood samples. The PEGDA/SA composite hydrogel enabled fast separation of small molecules from the complex blood matrix, while the plasmonic SERS substrate allowed for the sensitive detection of target molecules. 3D printing was employed to integrate the hydrogel membrane and the SERS substrate into a systematic device. The sensor achieved highly sensitive detection of dopamine in whole blood samples with a limit of detection down to 1 nM. The whole detection process from sample preparation to SERS readout can be finished within 5 min. Due to the simple operation and rapid response, the device shows great potential in point-of-care diagnosis and the monitoring of neurological and cardiovascular diseases and disorders.

## 1. Introduction

Neurotransmitters are chemical messengers released by neurons, glial cells, and platelets. They carry, boost, and balance signals across nerve cells and target cells throughout the body, such as glands and muscles [1,2]. There are hundreds of neurotransmitters in the human body which can be classified into monoamines, amino acids, peptides, purines, and gasotransmitters. The abnormal changes of these neurotransmitters in body fluids are strongly associated with mental disorders and neurodegenerative and cardiovascular diseases [3,4,5]. Therefore, the detection of neurotransmitters not only acts as the diagnostic tool for clinical diseases, but also advances the understanding of the biological processes related to these disorders [6,7,8]. Conventional techniques, such as chromatography [9] and mass spectrometry (MS) [10], require sophisticated operation and have a long processing time. Modern optical and electrochemical methods greatly simplify the sensing system while maintaining the selectivity and sensitivity of detection using recognition molecules including antibodies and aptamers [11,12]. Nevertheless, they still require a long incubation time and tedious sample preparation, which limits the applications in a point-of-care setting.

Surface enhanced Raman spectroscopy (SERS) has become a popular analytical tool for point-of-care biochemical analysis due to its high sensitivity and fast readout [13,14,15]. The SERS approach enables rapid and straightforward identification of target molecules without additional labels. It has been widely used for food safety [16], environmental monitoring [17], disease diagnosis [18], and drug analysis [19]. However, for the detection in biological fluids, the label-free SERS method suffers from interference in complex physiological environments [20]. Thus, to achieve the point-of-care detection of neurotransmitters in body fluids, it is desirable to develop an integrated device which can accomplish sample preparation and SERS sensing in a single platform.

For the label-free SERS detection of target molecules in blood, it is important to reduce the interference from the sample matrix. In a typical diagnostic lab, the target molecules are separated from whole blood using a centrifuge. In recent decades, the development of microfluidic technology has made it possible to achieve on-chip blood cell separation using hydrodynamic forces [21], magnetics [22], electrophoresis [23], microstructures [24], etc. However, the fabrication of these microfluidic chips relies on large facilities and increases the complexity and cost. To fit the point-of-care setting, there is always a need for developing simpler sensing systems with easy operation and low cost for end-users.

In this work, a 3D-printed SERS device was developed for the point-of-care analysis of neurotransmitters in whole blood. On the one hand, polyethylene glycol diacrylate (PEGDA)/sodium alginate (SA) composite hydrogels were used as filters for the rapid separation of blood cells and large molecules from whole blood, which enabled sample preparation without laboratory instruments. On the other hand, the plasmonic SERS substrate was fabricated with gold@silver core–shell nanorods (Au@Ag NRs) for the highly sensitive detection of neurotransmitters. A 3D-printed framework was employed to integrate the hydrogel membrane and the plasmonic SERS substrate into a single device, in which the hydrogel membrane and the SERS substrate were incorporated into the top and the bottom layer of the framework, respectively. To evaluate the performance of this device, the sensitivity of the SERS substrate and the filtration efficiency of the hydrogel membrane were tested. Dopamine was used as a representative neurotransmitter. Quantitative analysis of the neurotransmitters in whole blood was performed to evaluate the limit of detection in a physiological environment.

## 2. Materials and Methods

### 2.1. Materials

L-Ascorbic acid (AA), sodium hydroxide (NaOH), 2-hydroxy-4′-(2-hydroxyethoxy)-2-methylpropiophenone (Irgacure 2959, 98%), and decyl alchol (n-decanol) were purchased from Shanghai Titan Scientific Co., Ltd., Shanghai, China. Hexadecyltrimethylammonium bromide (CTAB), polyethylene glycol diacrylate (PEGDA, Mn = 700), and silver nitrate (AgNO_3_) were purchased from Sigma Aldrich. Sodium chloride (NaCl) was purchased from Shanghai Qiangshun Chemical Co., Ltd., Shanghai, China. Hydrogen tetrachloroaurate (III) trihydrate (HAuCl_4_·3H_2_O) was purchased from Alfa Aesar. Hydrochloric acid (HCl, 37%) was purchased from Kunshan Jincheng Reagent Co., Ltd., Kunshan, China. Sodium alginate (SA) and sodium borohydride (NaBH_4_) were purchased from Aladdin Biochemical Technology Co., Ltd., Shanghai, China. Hydrogen Peroxide (H_2_O_2_, 30%), Ethanol and Sulfuric acid (H_2_SO_4_, 98%) were purchased from Sinopharm Chemical Reagent Co., Ltd., Shanghai, China. Calcium chloride anhydrous (CaCl_2_) was purchased from Nanjing Chemical Reagent Co., Ltd., Nanjing, China. (3-Mercaptopropyl) trimethoxysilane (MPTMS) was purchased from Shanghai MackLin Biochemical Co., Ltd., Shanghai, China. 184 Silicone Elastomer Base (elastomer base) and 184 Silicone Elastomer Curing Agent (elastomer curing agent) were purchased from Dow Chemical Company, Midland, MI, USA. Human blood serum was provided by Jiangsu Province Hospital.

### 2.2. Synthesis of Au@Ag NRs

Au nanorods (Au NRs) were synthesized using a previously published 3-step method where n-decanol was introduced to assist tuning the final dimensions [25]. Briefly, first, 200 μL of 0.05 M HAuCl_4_ and 100 μL of AA solution were added to 20 mL of a 50 mM CTAB and 13.5 mM n-decanol solution under vigorous stirring. A transparent solution was obtained instantly and the reaction was continued for another 2 min. Then, 800 μL of an ice-cold and freshly prepared 0.02 M NaBH_4_ solution was injected rapidly at 25–28 °C, resulting in a brown seed solution. Second, 240 μL of 0.01 AgNO_3_, 2.1 mL of 1 M HCl, 300 μL of 0.05 M HAuCl_4_, and 390 μL of 0.1 M AA were added to 30 mL of a 50 mM CTAB and 13.5 mM n-decanol solution at 25 °C. Then, 1.8 mL of the seed solution was injected under gentle stirring for 30 s. After at least 4 h of growth in an undisturbed condition, the solution changed gradually from colorless to dark brown. The obtained anisotropic seeds were purified via centrifugation at 15000–17000 rpm for 30 min, and then redispersed with 2 mL of 10 mM CTAB solution. To purify the solution, another two centrifugations were performed under the same condition. Third, 150 μL of 10 mM AgNO_3_, 100 μL of 0.05 M HAuCl_4_, and 80 μL of 0.1 M AA solutions were added under stirring to 10 mL of 50 mM CTAB and 11 mM n-decanol solution at 28 °C, followed by adding 400 μL of 1 M HCl, and finally, 250 μL of the small anisotropic seed solution was injected to acquire Au NRs. After at least 4 h of reaction, a dark purple solution was obtained. The obtained Au NRs were purified by centrifugation twice at 6000 rpm for 10 min, and subsequently redispersed in 2 mL of 10 mM CTAB solution. Au@Ag NRs were prepared by adding 130 μL of 0.1 M AA, 50 μL of 15 mM AgNO_3_, and 240 μL of 0.1 M NaOH to 2 mL of the prepared Au NRs solution under intense stirring at 25–30 °C.

### 2.3. Fabrication of the SERS Substrate

Glass slides were immersed in freshly prepared piranha solutions (98% H_2_SO_4_: 30% H_2_O_2_, 3:1 *v*/*v*) for at least 1 h and then rinsed with a large volume of deionized water. After being rinsed repeatedly with deionized water and dried with argon gas three times alternately, the glass slides were immersed in a MPTMS (10%) solution at room temperature for 4 h to modify its surface with sulfur bonds. Then, they were washed with pure ethanol, dried with argon gas three times and dried at 60 °C for another 40 min. Subsequently, the Au@Ag NRs were centrifuged at 5000 rpm for 5 min and then redispersed in 20 mL of 8 mM NaCl solution, giving rise to the assembly of a high density of NRs. Afterwards, the uniform SERS substrate was obtained by immersing the glass slides with the Au@Ag NRs solution for 12 h at room temperature before use.

### 2.4. Synthesis of Hydrogels

A mixture of SA (2%), PEGDA (40%), and the photoinitiator (Irgacure 2959, 1.2% *w*/*w*) was prepared as the hydrogel precursor solution under vigorous stirring at room temperature. Subsequently, the mixture was degassed under vacuum for 1 h to avoid the creation of internal defects larger than the mesh size during polymerization. The hybrid hydrogel was formed via an in situ precipitation method. First, the ultraviolet (UV) lamp (365 nm) with the energy density of 400 mW/cm^2^ was utilized to induce the crosslinking of PEGDA hydrogel in the solution. After that, the solidified hydrogel was immersed in CaCl_2_ (2%) solution for 1 h to facilitate the ion exchange between Na^+^ and Ca^2+^ in sodium alginate. Finally, in order to remove excess ions, the hydrogel was soaked in deionized water for 2 h, and PEGDA/SA hydrogel was obtained.

### 2.5. Printing the Framework of the Device

The framework of the device in this study was printed using a 3D printing system. The framework was composed of two layers. The top layer was supported by the network between the two layers, which served as a supporting surface to prevent deformation and breakage of the hydrogel filter membrane under negative pressure. The bottom layer was designed to place the SERS substrate on. A hole was designed on the left side to enable vacuum extraction through an attached syringe, which generated a pressure differential across the hydrogel filter. Specifically, it created negative pressure within the chamber, driving fluid penetrating through the hydrogel membrane, ultimately giving rise to separation and filtration. After printing, the device was immersed in ethanol and cleaned under ultrasonic oscillation for 20 min to remove any impurities. Then, the device was dried in an oven at 40 °C for 10 min prior to use.

### 2.6. Integration of the Sensor

In this experiment, a syringe, SERS substrate, hydrogel membrane, and the framework of the device were combined together to integrate the sensor. In a standard procedure, the syringe injection port was connected to the left-side hole and the SERS substrate was fixed onto the bottom of the chamber. Additionally, to improve the airtightness of the chamber, elastomer base and elastomer curing agent were mixed with a 10:1 weight ratio to acquire polydimethylsiloxane (PDMS), which was filled at the entrance of the SERS substrate and the vacuum extraction hole of the syringe, followed by incubation in an oven at 50 °C for 10 min. Finally, to obtain the ultimate sensor, the prepared hydrogel membrane was attached to the supporting layer located in the middle of the chamber.

### 2.7. Counting of Blood Cells

The blood cells were quantified using a hemocytometer. Briefly, 0.1 μL of each sample was loaded onto both sides of a hemocytometer counting chamber. After incubation for a few minutes to allow the cells to settle, the number of cells within multiple grid squares were counted and recorded under a microscope.

### 2.8. SERS Detection of Neurotransmitters

Different concentrations of dopamine (from 1 nM to 1 mM) were added to the blood samples. Each sample was added onto the filter membrane. Then, a syringe was used to extract the vacuum from the left-side hole. The negative pressure inside the chamber induced the penetration of blood through the hydrogel filter. After around 3 min, the droplets of sample were deposited onto the SERS substrate at the bottom. Finally, the substrate was taken out of the device for SERS measurement.

### 2.9. Instruments and Measurement

The synthesis of Au NRs and Au@Ag NRs were monitored using UV-vis (L9, INESA, Shanghai, China). Transmission electron microscope (TEM) images were obtained with an electron microscope (Tecnai 12, Philips, Amsterdam, The Netherlands) operating at 120 kV. A high-speed centrifuge (2-16 PK, Sigma, Osterode, Germany) was used for sample purification. The original rigid platform framework was printed using a microscale 3D printing system with high-precision manufacturing capabilities (NanoArch P150, BMF, Chongqing, China). A scanning electron microscope (S4800, Hitachi, Tokyo, Japan) was employed to obtain the morphology of the cross-section of the hydrogel. SERS signals were collected with a Raman spectrometer (T64000, Horiba, Kyoto, Japan). A He-Ne laser with 633 nm radiation was used for excitation and the laser power at the sample position was 5 mW. All the spectra here were the result of an accumulation of 10 s.

## 3. Results and Discussions

### 3.1. Design and Principle of the Device

**Figure 1:**
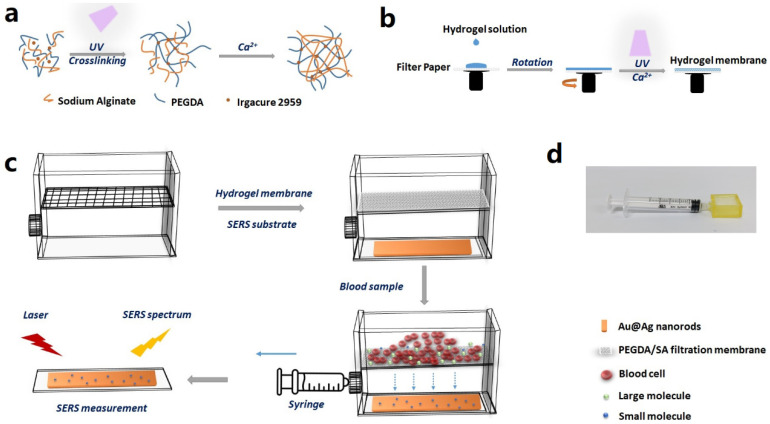
(**a**) Schematic illustration of the mechanism for synthesizing PEGDA/SA composite hydrogel. (**b**) Schematic illustration of the protocols for fabricating a hydrogel membrane. (**c**) Schematic overview of the device and the working principle. (**d**) A photo of the device.

Figure 1a shows the protocols for synthesizing PEGDA/SA composite hydrogel. First, the mixture of PEGDA, SA, and Irgacure 2959 was exposed to the UV lamp. The Irgacure absorbed the energy of the light and the PEGDA rapidly solidified into a crosslinked gel. Second, the SA gel was generated in the presence of Ca^2+^ ions, and rapidly formed an interpenetrating double network within the gel structure, thus obtaining the PEGDA/SA composite hydrogel.

Figure 1b shows the protocols for fabricating a hydrogel membrane. The filtration membrane was prepared by the spincoating of the PEGDA/SA precursor solution. Specifically, a wet filter paper (cellulose membrane) was adsorbed onto the rotating platform, and then the hydrogel solution was dropped onto the center of the platform. After spincoating, a UV lamp was utilized to cure the PEGDA. When the solution on the surface solidified to PEGDA hydrogel, it was immersed in a CaCl_2_ solution for ion exchange. During this process, a uniform and stable PEGDA/SA hydrogel network was obtained.

Figure 1c provides a schematic overview of the device and the working principle. First, a 3D printer was employed to fabricate a framework for the device, which was composed of two layers, with a network between the top and bottom layer. Second, the top layer was filled with the hydrogel membrane, which was supported by the network. The bottom layer was inserted with the prepared SERS substrate assembled with Au@Ag NRs. There was an air-filled hollow space between the hydrogel membrane and the SERS substrate. Third, the blood sample with neurotransmitters was introduced to the top layer of the device. A syringe was used to withdraw air from the air-filled hollow space in the bottom layer, so that the negative pressure induced the penetration of blood through the hydrogel membrane. The blood cells and the large molecules were prevented by the hydrogel filter, while small molecules passed through the membrane to the bottom layer, which was embedded with the SERS substrate. Finally, the SERS substrate was taken out of the device and exposed to the laser for SERS measurement. By acquiring the SERS spectrum, quantitative detection of the neurotransmitters was facilitated.

### 3.2. Characterization of Nanoparticles and Nanosubstrates

**Figure 2:**
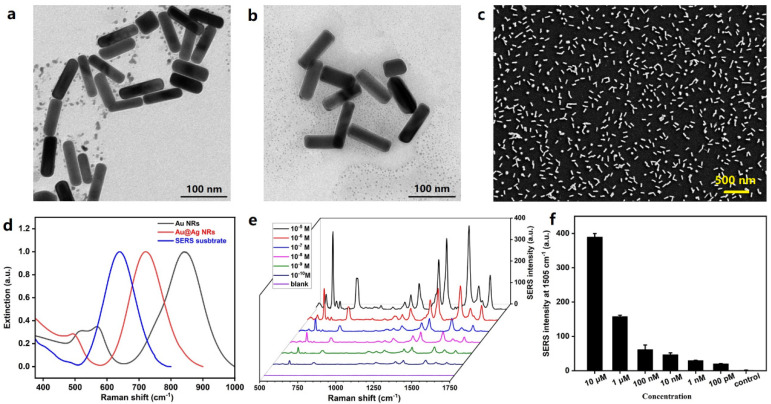
Characterization of plasmonic nanoparticles and nanosubstrate. (**a**) TEM image of Au NRs. (**b**) TEM image of Au@Ag NRs. (**c**) SEM image of the SERS substrate. (**d**) Extinction spectra of Au NRs, Au@Ag NRs, and the SERS substrate. (**e**) Concentration-dependent SERS spectra for different concentrations of R6G. (**f**) Histogram plot of the peak intensity at 1505 cm^−1^ as a function of the concentration of R6G.

Plasmonic nanoparticles were fabricated for SERS sensing. To increase the sensitivity, Au@Ag NRs, which demonstrated superior SERS activity, were used in this device. The core–shell nanorods were obtained by coating Au NRs with silver shell. Here, the method by González-Rubio et al. was employed to fabricate Au NRs with a high aspect ratio [25]. The TEM image in Figure 2a indicates an average length, width, and aspect ratio of 84.3 nm, 23.8 nm, and 3.618, respectively. After silver coating, the average length and width increased to 91.9 and 31.1 nm, respectively, and the average aspect ratio was 3.04 (Figure 2b). It can also be observed from the extinction spectra in Figure 2d (black and red curves) that the longitudinal SPR band blue-shifted from 850 nm to 721 nm, which confirmed the presence of a silver shell. The Au@Ag NRs were assembled onto a glass slide through chemical bonding. It is reflected in the SEM image in Figure 2c that the nanorods were mostly evenly distributed onto the substrate, with a density of around 44/μm^2^. Figure 2d (blue curve) shows the extinction spectrum of the SERS substrate. It can be observed that the SPR band further blue-shifted to 639 nm when the nanoparticles were transferred from liquid solutions to solid substrate, which can be explained by the decreased dielectric constant of their surrounding environment. Thus, a He-Ne laser with an emission wavelength of 633 nm, which is close to the SPR wavelength of Au@Ag NRs at 639 nm, was used for Raman excitation. Using an R6G molecule as the Raman reporter, the enhancement factor of the SERS substrate was estimated to be 10^7^ (Figure S1). Quantitative analysis was also performed to evaluate its SERS performance (Figure 2e). As shown in Figure 2f, the SERS intensity was positively correlated with the concentration of R6G, reaching a limit of detection down to 100 pM.

### 3.3. Characterization of Polymer Filters

**Figure 3:**
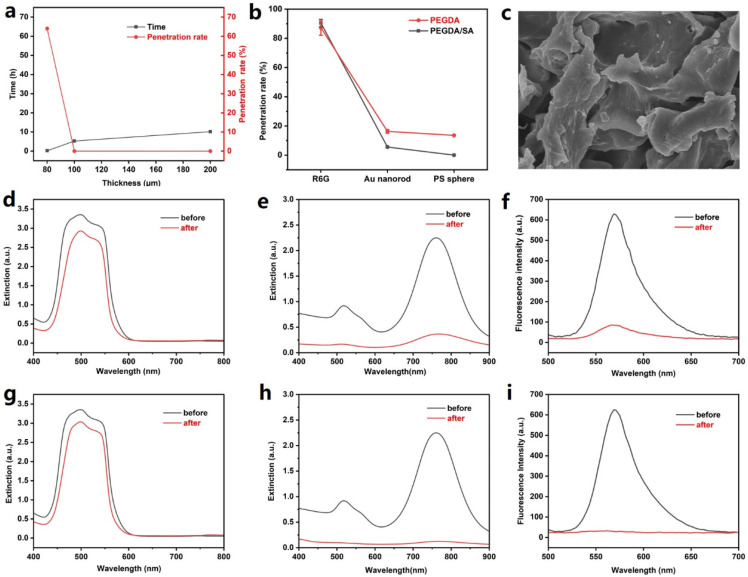
Characterization of the hydrogel membranes. (**a**) Plot of the total time and penetration rate of PS spheres (in 50 mL water) for hydrogel membranes with different thicknesses. (**b**) Plot of penetration rates of R6G, Au nanorod, and PS sphere using PEGDA and PEGDA/SA composite hydrogel membrane. (**c**) SEM image of the PEGDA/SA hydrogel. The scale bar indicates a length of 50 μm. (**d**) Extinction spectra of R6G solution before and after passing through the PEGDA hydrogel membrane. (**e**) Extinction spectra of Au nanorod solution before and after passing through the PEGDA hydrogel membrane. (**f**) Fluorescence spectra of PS sphere (labelled with Nile red) solution before and after passing through the PEGDA hydrogel membrane. (**g**) Extinction spectra of R6G solution before and after passing through the PEGDA/SA hydrogel membrane. (**h**) Extinction spectra of Au nanorod solution before and after passing through the PEGDA/SA hydrogel membrane. (**i**) Fluorescence spectra of PS sphere (labelled with Nile red) solution before and after passing through the PEGDA/SA hydrogel membrane.

The blood filter was fabricated using hydrogels. The porous matrix of hydrogel served as a barrier to block blood cells and macromolecules while allowing the penetration of micromolecules. To optimize the performance of the hydrogel filter, the thickness of the hydrogel membrane was carefully controlled. Hydrogel membranes with a thickness of around 80, 100, and 200 μm, respectively, were fabricated and 50 mL of solutions containing PS spheres were used as the test solutions. It can be observed from Figure 3a that the penetration rates were around 0.2% when the thicknesses of the membranes were 100 and 200 μm. However, this rate increased to around 60% when the thickness decreased to 80 μm, which indicated severe damage to the membrane. Hence, the thickness of the hydrogel membrane was set to 100 μm in the subsequent experiments.

To evaluate the performance for blood filtration, R6G, Au nanorods (with an average length and diameter of 20 and 8 nm, respectively), and PS spheres (with a diameter of 50 nm) were used to simulate biomolecules of different sizes. The penetration rates of these three substances were obtained by comparing their concentrations before and after passing through the filter membrane. Here, extinction spectroscopy was employed to characterize the concentrations of R6G and gold nanorods, while fluorescence spectroscopy was used to characterize the concentration of PS spheres. As shown in Figure 3d–f, the spectra of these three substances before and after passing through the membrane were acquired, and the penetration rates of R6G, Au nanorod, and PS sphere were calculated to be 87.3%, 16.3%, and 13.6%, respectively (Figure 3b, red curve). The results demonstrated the size selectivity of the hydrogel.

To improve the performance of the hydrogel filters, PEGDA/SA composite hydrogel was fabricated and the penetration rates were also tested (Figure 3b, black curve). SEM imaging was used to characterize the morphology of the PEGDA/SA polymer, which exhibited the porous structure of the hydrogel (Figure 3c). It is reported that the addition of SA in PEGDA hydrogel would decrease the pore size while increasing the gel strength [26]. It can be observed that R6G molecules exhibited a penetration rate of 90.3% (Figure 3g), which is close to that using PEDGA hydrogel. Meanwhile, the penetration rate of Au NRs decreased to 5.6% (Figure 3h), which can be explained by the higher density of crosslinking and increased strength of the hydrogel. The results in Figure 3i indicate that the PEGDA/SA efficiently blocked the PS spheres, with a penetration rate down to 0.048%. It can be concluded from the comparison that the PEGDA/SA composite hydrogel effectively blocked large particles while permitting the penetration of small molecules, which would be better suited for analyzing small molecules in whole blood samples.

### 3.4. Neurotransmitters Sensing in Whole Blood

**Figure 4:**
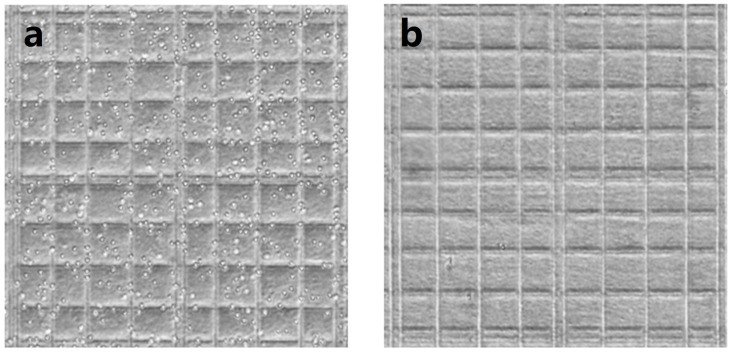
Bright field image of whole blood samples on a cell counter plate (**a**) before and (**b**) after passing through the hydrogel filter.

Neurotransmitters act as important indicators of neurodegenerative and cardiovascular disease. To achieve rapid analysis of neurotransmitters in blood, PEGDA/SA hydrogel was employed as a barrier to block blood cells and macromolecules, while allowing small molecules, including neurotransmitters, to pass through. Figure 4 shows the bright field image of blood samples before and after filtration; it can be observed that the PEGDA/SA hydrogel efficiently removed blood cells from the blood sample. No blood cells were observed in the bright field image.

**Figure 5:**
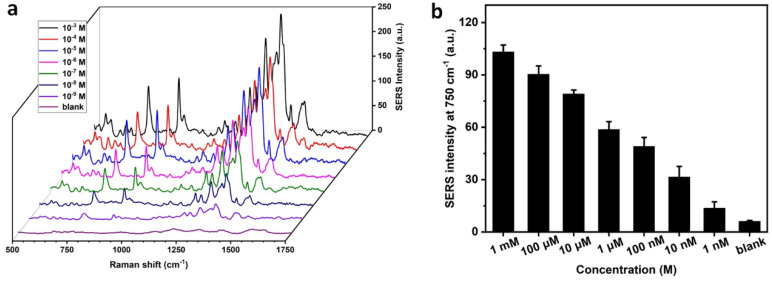
Quantitative analysis of dopamine in whole blood. (**a**) Concentration-dependent SERS spectra of dopamine in whole blood samples. (**b**) Plot of SERS intensity at 750 cm**^−^**^1^ as a function of the concentration of dopamine.

Dopamine was employed as a representative analyte. Different concentrations of this neurotransmitter were added to blood samples. Figure 5a shows the concentration-dependent SERS spectra of dopamine. The dopamine molecule was characterized by the Raman bands at 750 cm^−^^1^, which was attributed to in-plane bending vibration arising from the phenolic ring [27]. By plotting the relationship between the peak intensity and the concentration (Figure 5b), it can be concluded that the LOD for dopamine detection in whole blood was 1 nM. The whole detection from sample preparation to SERS readout can be finished within 5 min, making it a potentially powerful tool for point-of-care disease diagnosis and monitoring. Although the current SERS measurement was performed in the lab, the portable Raman spectrometer provides a perfect solution to promote its future application in resource-limited areas. In this work, dopamine was manually added to blood samples to mimic the clinical samples. It is worth noting that whole blood contains hundreds of small molecules which may lead to band overlap in the analysis of real clinical samples. Nevertheless, with the rapid development of artificial intelligence, the SERS technique could potentially be applied to real clinical analysis in the future.

## 4. Conclusions

We have developed a SERS composite hydrogel device which achieved rapid and straightforward analysis of neurotransmitters in whole blood. The device is composed of a hydrogel membrane, a plasmonic SERS substrate, and a 3D-printed framework. On the one hand, the porous nanostructure of the PEGDA/SA hydrogel served as a filter to block blood cells and large molecules while allowing the penetration of small molecules. On the other hand, the SERS substrate assembled with Au@Ag NRs acted as a signal amplifier to enhance the Raman signatures of the neurotransmitters. Quantitative analysis of a representative neurotransmitter, dopamine, was achieved, with a limit of detection down to 1 nM in whole blood samples. The response time from ‘sample in’ to ‘answer out’ was 5 min. Since the developed device is simple to manufacture, easy to use, fast to respond, and low in cost, it is expected to find enormous applications in point-of-care disease diagnosis and monitoring.

## Data Availability

Data available from the corresponding authors upon reasonable request.

## Supplementary Materials

The following supporting information can be downloaded at: https://www.mdpi.com/article/10.3390/bios13060611/s1, Figure S1: Raman and SERS spectra of R6G.
