# Supplementary material for: A SERS Composite Hydrogel Device for Point-of-Care Analysis of Neurotransmitter in Whole Blood
Source: Biosensors (Basel). 2023 Jun 2;13(6):611. doi: 10.3390/bios13060611 (PMC10295853; doi:10.3390/bios13060611)
Supplement: Supplementary file 1 [file biosensors-13-00611-s001.zip › biosensors-2406860-supplementary.pdf]

Supplementary Material

A SERS Composite Hydrogel Device for Point-of-Care Analysis of Neurotransmitter in Whole Blood

Lei Wu, Xuefeng Liu, Shenfei Zong, Zhuyuan Wang * and Yiping Cui *

Advanced Photonics Center, School of Electronic Science and Engineering, Southeast University, Nanjing 210096, China; lwu@seu.edu.cn (L.W.); 220201515@seu.edu.cn (X.L.); sfzong@seu.edu.cn (S.Z.)

* Correspondence: wangzy@seu.edu.cn (Z.W.); cyp@seu.edu.cn (Y.C.)

1. Calculation of the SERS enhancement factor

To calculate the SERS enhancement factor of the substrate, the Raman and SERS spectra of 10^{-4} M and 10^{-7} M R6G solution were acquired, respectively. As shown in Fig.S1, the SERS and Raman intensity ratio is around 10^4. Hence, the SERS enhancement factor is estimated to be 10^7.

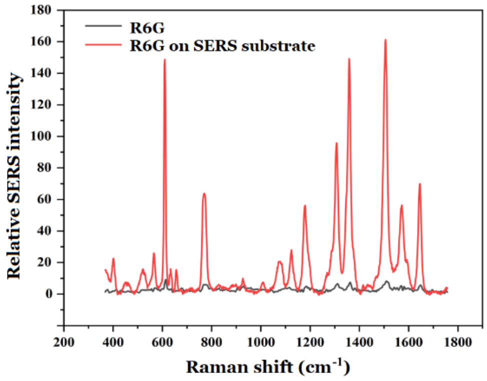

Figure S1. Raman and SERS spectra of R6G.
